# Supplementary material for: Hypoxia Decreases Diagnostic Biomarkers for Aspergillosis In Vitro
Source: J Fungi (Basel). 2019 Jul 11;5(3):61. doi: 10.3390/jof5030061 (PMC6787614; doi:10.3390/jof5030061)
Supplement: Supplementary file 1 [file jof-05-00061-s001.pdf]

Supplementary Materials: Hypoxia Decreases Diagnostic Biomarkers for Aspergillosis In Vitro

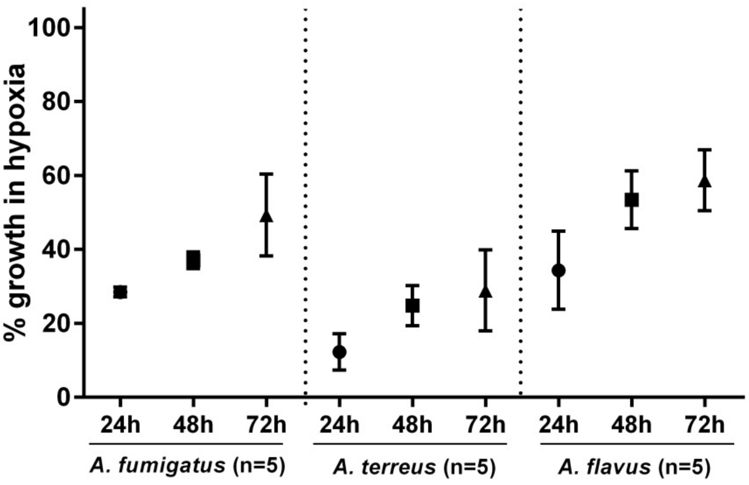

Figure S1. Hypoxia led to reduced biomass of *Aspergillus* species. Strains were grown in liquid $RPMI_{1640}$ containing 2% glucose at 37 °C under normal oxygen and hypoxic growth conditions for 24 h, 48 h and 72 h. Fungal growth (%) represents percentage of biomass from hypoxic cultures normalized to normoxic growth. For calculations average biomass of all five tested strains was used.

Table S1. Release of diagnostic biomarkers during in vitro growth of different *Aspergillus* spp. in $RPMI_{1640}$ medium under normal and hypoxic growth conditions. Amount of GM and BDG release was normalized to biomass (dry weight). Multiple t- test (graph pad prism software) was used for statistical analysis of biomarker release in normoxic conditions versus hypoxic conditions, p- values ≥ 0.05 were considered statistically different.

		µg GM/mg dry weight					µg BDG/mg dry weight				
		normoxia		hypoxia			normoxia		hypoxia		
species	time (h)	mean	SD	mean	SD	p- value	mean	SD	mean	SD	p- value
A. fumigatus	24h	3.27	0.14	11.51	2.96	0.001	0.09	0.07	0.27	0.16	0.04
(n=5)	48h	7.58	3.60	11.36	3.86	0.08	0.14	0.05	0.16	0.06	0.53
	72h	12.10	3.04	10.15	2.50	0.29	0.11	0.03	0.14	0.07	0.44
A. terreus	24h	8.96	1.87	26.89	10.22	0.05	0.16	0.03	0.76	0.52	0.05
(n=5)	48h	21.40	4.14	15.94	4.67	0.47	0.23	0.13	0.24	0.08	0.52
	72h	18.11	2.16	21.74	5.77	0.23	0.19	0.09	0.19	0.07	0.99
A. flavus	24h	11.41	0.84	20.55	6.01	0.16	0.08	0.02	0.30	0.12	0.13
(n=5)	48h	8.56	2.78	7.57	0.98	0.59	0.10	0.07	0.23	0.06	0.23
	72h	14.40	3.12	9.93	0.31	0.18	0.14	0.06	0.15	0.07	0.76

GM; galactomannan; BDG; β-(1,3)-glucan; SD; standard derivation.
